# Low-fidelity simulation models in urology resident’s microsurgery training

**DOI:** 10.1590/acb386523

**Published:** 2023-12-01

**Authors:** Luís Otávio Amaral Duarte Pinto, Renata Cunha Silva, Lívia Guerreiro de Barros Bentes, Herick Pampolha Huet de Bacelar, Kátia Simone Kietzer

**Affiliations:** 1 Universidade Estadual do Pará – Department of Integrated Health – Belém (PA), Brazil.; 2 Universidade Estadual do Pará – Department of Morphophysiology Applied to Health – Belém (PA), Brazil.; 3 Universidade Estadual do Pará – Experimental Surgery Laboratory – Belém (PA), Brazil.

**Keywords:** Medical Education, Simulation Training, Printing, Three-Dimensional, Urology, Microsurgery

## Abstract

**Purpose:**

To evaluate the gain of microsurgical skills and competencies by urology residents, using low-fidelity experimental models.

**Methods:**

The study involved the use of training boards, together with a low-fidelity microsurgery simulator, developed using a 3D printer. The model consists in two silicone tubes, coated with a resin, measuring 10 cm in length and with internal and external diameters of 0.5 and 1.5 mm. The support for the ducts is composed by a small box, developed with polylactic acid. The evaluation of the gain of skills and competencies in microsurgery occurred throughout a training course consisting of five training sessions. The first sessions (S1-S4) took place at weekly intervals and the last session (S5) was performed three months after S4. During sessions, were analyzed: the speed of performing microsurgical sutures in the pre and post-training and the performance of each resident through the Objective Structure Assessment of Technical Skill (OSATS) and Student Satisfaction Self-Confidence tools in Learning (SSSCL).

**Results:**

There was a decrease in the time needed to perform the anastomosis (p=0.0019), as well as a progressive increase in the score in the OSATS over during sessions S1 to S4. At S5, there was a slightly decrease in performance (p<0.0001), however, remaining within the expected plateau for the gain of skills and competences. The SSSCL satisfaction scale showed an overall approval rating of 96.9%, with a Cronback alpha coefficient of 83%.

**Conclusions:**

The low-fidelity simulation was able to guarantee urology residents a solid gain in skills and competencies in microsurgery.

## Introduction

Urology is the medical specialty responsible for treating problems related to the urinary tract of both sexes and diseases of the male genital system. It has a great interface with several other areas of medicine, covering the care of men, women, children, and the elderly. In Brazil, to become a urologist, every health professional must have a degree in medicine and specialization (medical residency) in general surgery and urology, totaling approximately 11 years of studies to be able to treat, clinically or surgically, the patients^1^.

During the medical residency in urology, aspirants do medical appointments, accompany hospitalized patients, and learn to perform surgeries and diagnostic tests for a series of diseases, both benign and malignant, involving the kidneys, ureters, bladder, urethra, prostate, adrenals, testes, epididymis, and penis. Among the most prevalent pathologies, urinary calculi, prostatic hyperplasia, sexual dysfunctions, male infertility, etc. stand out^1^^,^ 2.

Unfortunately, the learning of urology residents linked to the Unified Health System (SUS) has been the target of criticism by a considerable part of the health education institutions in the country and by the Brazilian Society of Urology itself. The lack of investment in teaching hospitals has not kept pace with the growth of innovative technologies for the treatment of urological diseases. Therefore, most residents have deficiencies in their training, having little contact with procedures considered commonplace for a good practice of their profession^3^. The main difficulties pointed out by Brazilian urology residents involve learning about surgeries that require orthoses, prostheses, and special materials, such as endoscopic treatment of urinary calculi, laparoscopic and robotic surgeries, and the application of urological microsurgery^4^.

Microsurgery consists of performing surgical procedures that require the aid of image magnification, through magnifying glasses or optical microscopes. In urology, its use is well established by those professionals who are dedicated to the management of male infertility, the so-called andrologists. In this area, the surgeries that most commonly use this type of equipment are varicocele correction and vasovasostomy, also known as vasectomy reversal^5^.

Varicocele is considered the main cause of infertility in men, which can be treated surgically. This disease is characterized by dilation and loss of functionality of the valve mechanism of the spermatic cord veins, causing blood reflux into the testicle. This greater venous return promotes a change in the temperature of the testicle, leading to an increase in the production of free radicals and consequent losses in the production and quality of spermatozoa^6^. Regarding vasectomy reversal, despite the scarcity of official epidemiological data, it is assumed that 6% of men undergoing this sterilizing surgery seek to regain their fertility at some point in their lives. Among the main reasons for this search, some life circumstances stand out, such as the death of children, divorces, new relationships, etc.^5^^,^ 7.

Both varicocele correction and vasovasostomy are considered minor surgeries, with quick recovery. Besides, despite the demand of an increasing number of patients, the training of a good part of urology residents in the country in these procedures is not enough, although this training has been satisfactorily offered by medical residency programs linked to the SUS^4^^,^ 5. These gaps in learning end up favoring a certain limitation of this future specialist in his/her area of expertise, making him/her an insecure and repressed professional, making it even more difficult for the public network to solve the main urological pathologies, given that the SUS ends up being the main entry point for these physicians in the labor market^8^^,^ 9.

Some health education institutions have been looking for alternatives to alleviate the difficulties encountered in training surgical residents. In this context, the use of simulators, or experimental training models, has been gaining increasing prominence, as a complementary method in the teaching of surgical specialties^10^. In microsurgery, for example, simulation has been encouraged for some years now, primarily encompassing the use of experimental animals and practice on cadavers. However, the legal difficulties for using bodies for educational purposes, as well as pressure from society to reduce animal experimentation and the restriction of its handling to a select number of research centers, have been encouraging the replacement of these types of training by use of synthetic models^11^^,^ 12.

The diffusion of artificial simulators, in the training of different medical specialties, occurs mainly due to the cheaper, improvement and popularization of three-dimensional (3D) printing technology. However, it is important to highlight that most of these 3D models described in the scientific literature are of low fidelity, meaning that many authors are still reluctant to use them routinely, especially regarding the quality of training that can be offered to beginners in surgical practices^10^.

Thus, observing a greater demand from men who seek infertility treatments, as well as the lack of training for urology residents in microsurgery and the increasingly common use of artificial training models, as an auxiliary instrument in medical education, the present study devised to evaluate the gain of microsurgical skills and competencies by urology residents, using low-fidelity experimental models.

## Methods

### Ethical aspects

The study was carried out at the Laboratory of Morphophysiology Applied to Health, at the Universidade Estadual do Pará (UEPA), obeying all ethical norms for research involving human beings, with its implementation approved by the Research Ethics Committee of the institution, through obtaining the Certificate of Presentation of Ethical Appreciation number 48382121.9.0000.5174.

### Low-fidelity simulators

For training in urological microsurgery, five training plates were used, together with a low-fidelity simulator for vasectomy reversal training, developed by the Laboratory of Experimental Surgery, in partnership with the Laboratory of Morphophysiology Applied to Health at UEPA.

**Figure 1:**
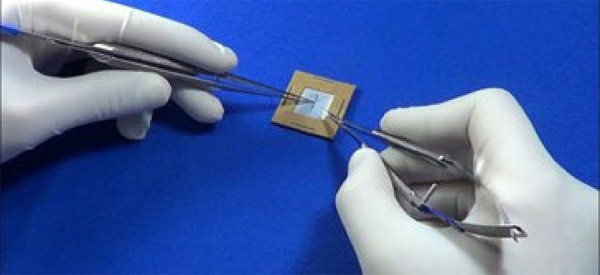
Training plate used to make sutures during pre- and post-training.

Source: Authors’ archive.

The training plates are 2 × 2 cm in size and were made with a snip of a latex glove, fixed between two square cardboard frames measuring 2 × 2 cm on each side, stapled together (Fig. 1).

**Figure 2:**
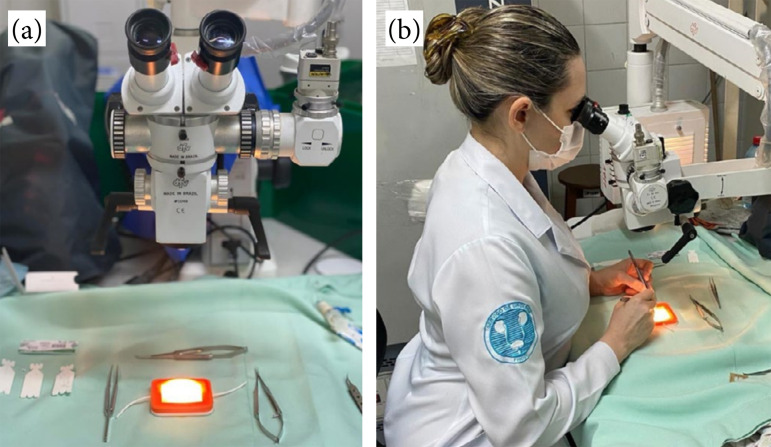
Microsurgery training using the low-fidelity model. **(a)** Training station with a three-dimensional simulator; **(b)** resident conducting training session with a low-fidelity simulator.

Source: Authors’ archive.

**Figure 3:**
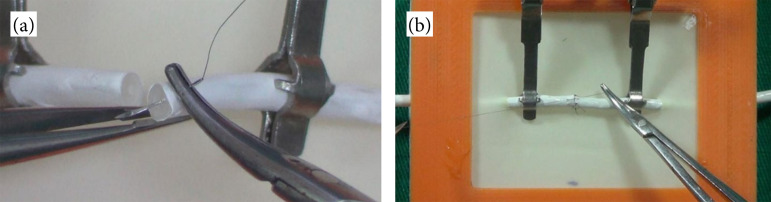
Creation of microsurgical anastomosis during the training session with a low-fidelity simulator built in a three-dimensional printing. **(a)** Stitch transfix; **(b)** microanastomosy.

Source: Authors’ archive.

The low-fidelity experimental model, in turn, basically consists of two translucent silicone tubes, attached to a support made with a 3D printer. The tubes are 10-cm long each, with an internal and external diameter of 0.5 and 1.5 mm, respectively, and were externally coated with a film of polyvinyl acetate (PVA) resin, white, enclosing an artificial vas deferens, with their respective histological layers (luminal, muscular, and adventitial mucosa). Figure 2 shows the training station and the posture during training. Figure 3 illustrates the low-fidelity simulators, as well as the performance of a microsurgical anastomosis using the device. The materials and description of the simulator developed using 3D printing can be found in more details in a previous publication^5^.

### Microsurgery training with models

To evaluate the performance of low-fidelity simulators as an auxiliary tool in teaching and learning in urological microsurgery, a study was conducted involving nine urology residents enrolled in two tertiary hospitals in Pará, linked to SUS. As inclusion criteria, all residents should agree with the work, signing the Informed Consent Form (TCLE), in addition to having completed at least the first year of medical residency (R1). Those residents who did not sign the TCLE and who were still attending the R1 of urology were excluded from the study.

The study participants were evaluated through their performance in the simulator, during the II Training Course in Urological Microsurgery promoted by UEPA. The course consisted of a first day of adaptation (S0), followed by four training sessions, with weekly intervals between them (S1, S2, S3 and S4) and an extra session (S5), which took place 12 weeks after S4. To ensure better use by residents, all training sessions were individualized.

During the setting (S0), residents should watch a theoretical video class, which demonstrated basic aspects of managing the microscope, positioning, operative technique, as well as an additional practical activity of microsurgical sutures on training plates, lasting half an hour. The remainder of the training course (S1 to S5) was based on suturing and anastomosing.

From S1 to S5, at the beginning and at the end of each session, the residents had to perform two microsurgical sutures on the training board, in which their time for making the knots was measured, called respectively “pre-training” and “post-training”. Each suture should consist of a double knot, followed by two simple knots.

The training itself lasted one hour and was developed through practice using the simulator made with 3D printing. Each participant should perform an anastomosis between the two silicone tubes, containing a total of eight sutures, half of which in the total plane (covering all its layers) and the rest in the partial plane (sparing its lumen), similar to the simplified vasovasostomy technique, advocated by Ramada-Benlloch et al.^13^. At the end of each training session, there was a moment of debriefing, in which the positive points of each participant were highlighted, as well as the main aspects that should be improved for subsequent training sessions.

The instruments used in the training were: Castroviejo microsurgical needle holder, 10-cm long and without rack; dissecting forceps, watchmaker type, straight and curved, 10-cm long; Castroviejo curved scissors, 10-cm long and Goldstein microspike approximating clamp. The thread used in making the sutures was nylon 8-0, 2 ¼ spatular needles, 0.65–30 cm, from Bioline. The anastomosis was performed with the aid of stereoscopic magnification, at least 10x, through a conventional optical microscope D. F. Vasconcelos.

### Assessment of the gain of skills and competences

**Figure 4:**
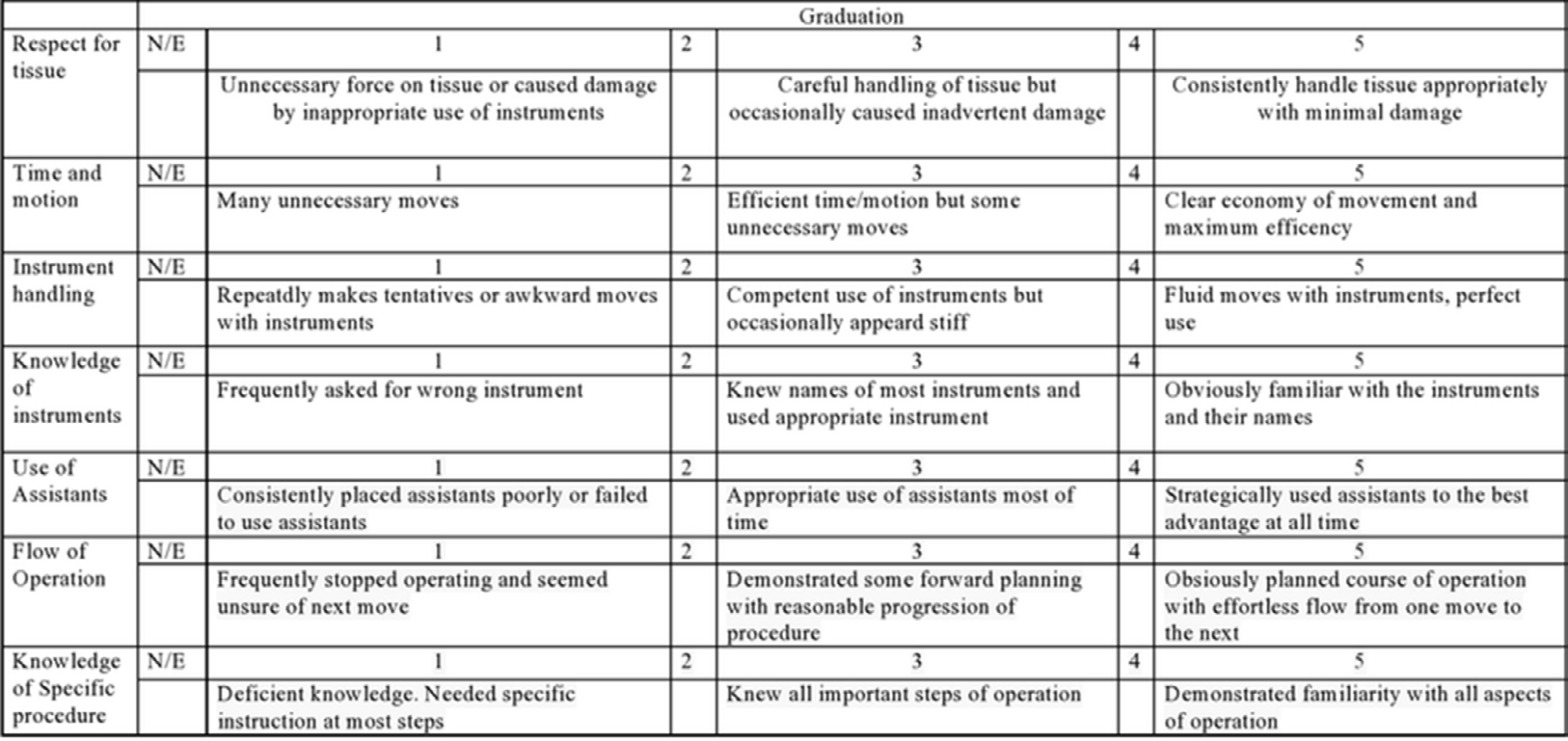
Objective structured assessment of technical skill global rating scale: Objective Structured Assessment of Technical Skill.

Source: Chevallay et al.^16^.

**Figure 5:**
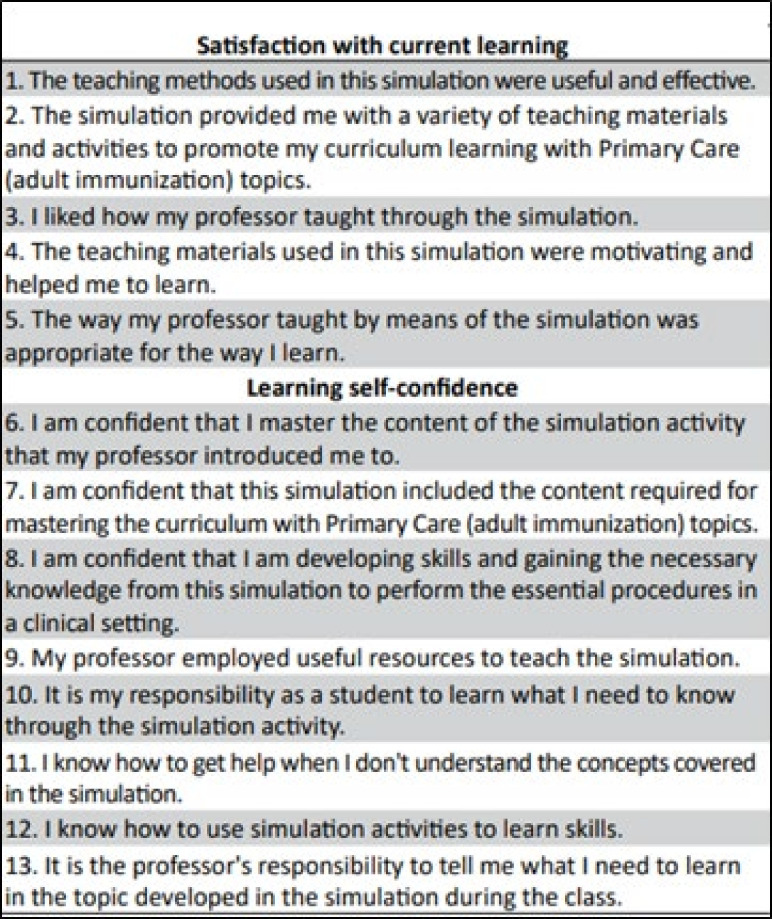
Student Satisfaction Scale and Self-Confidence in Learning.

Source: Costa et al.^17^.

To verify the acquisition of expertise in microsurgery, the recording of the time for making the sutures in the pre- and post-training period, throughout the five training sessions, was used, as well as performance analysis with the use of the simulator made in 3D printing, through the scale of global assessment called Objective Structured Assessment of Technical Skill (OSATS), containing seven aspects to be evaluated, with scores ranging from 1 to 5 for each item (Fig. 4). At the end of the course (S5), residents also performed a subjective assessment by completing a self-assessment questionnaire, on a five-point Likert scale, entitled Scale of Student Satisfaction and Self Confidence in Learning (SSSCL) (Fig. 5), containing 13 statements, divided into two domains: satisfaction with current learning (with five statements) and self-confidence in learning (with the remaining eight statements). All 13 sentences allowed the following response possibilities: completely disagree (1 point), partially disagree (2 points), neither agree nor disagree (3 points), partially agree (4 points), completely agree (5 points). Both assessment tools (OSATS and SSSCL) were used in their duly validated versions for the Portuguese language^14^^,^ 15.

### Statistical analysis

Data were collected and processed in Microsoft Excel and Word 2013 programs to create tables and graphs and subsequently submitted to statistical analysis using the BioEstat 5.4 program. Initially, the results obtained were verified using the Shapiro-Wilk’s normality test. To compare the parametric data, the analysis of variance (ANOVA) and paired Student’s t tests were used. Values of p ≤ 0.05 were considered statistically significant. For the SSSCL analysis, the scores obtained in each of the 13 statements were calculated, as well as their averages in each domain. To study the reliability and internal consistency of the questionnaire, Cronbach’s alpha index was used, with a coefficient value greater than 0.75, or 75%, being considered validated.

## Results

**Figure 6:**
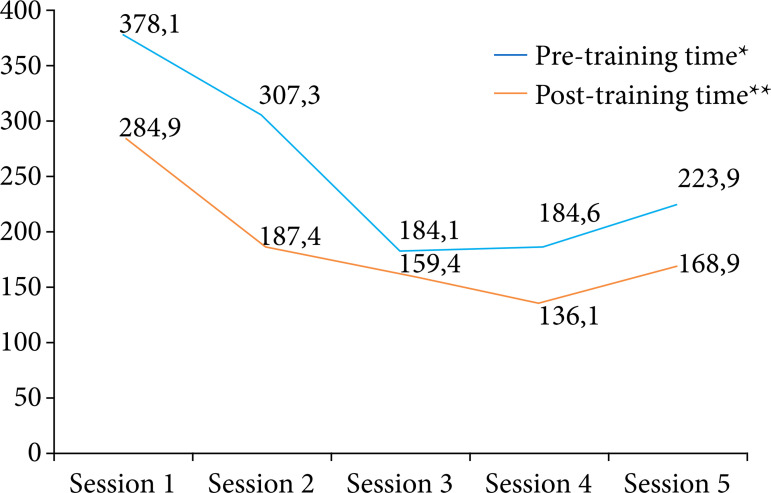
Average time, in seconds, of the sutures during pre- and post-training, throughout the training sessions.

In Fig. 6, the comparison of the average time of the participants to perform microsurgical sutures, before and after the training sessions (pre- and post-training), is observed. Analyzing the graph, it can be verified that, in all sessions, the sutures made during the post-training were faster than those of the pre-training, with p = 0.0019. In addition, it is possible to significantly verify a gradual improvement in the performance of the execution of microsurgical sutures, throughout the training sessions that took place at weekly intervals (S1 to S4), showing a decrease in performance later, in S5, which occurred three months after the last training, with p < 0.0001 and p < 0.0006, in pre- and post-training, respectively.

*Student’s t test: p = 0.0001; **analysis of variance: p = 0.0006. Source: Elaborated by the authors.

**Figure 7:**
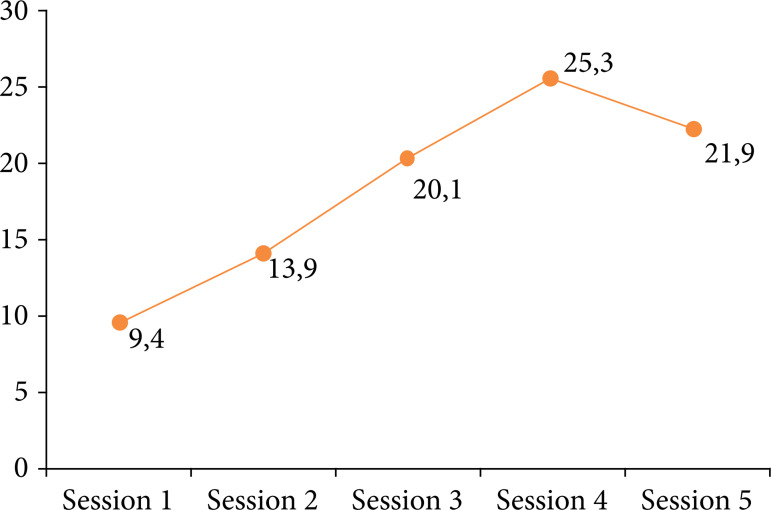
Objective Structure Assessment of Technical Skill average score throughout the training sessions*.

In turn, Fig. 7 is the graphic representation of the gain in skills and competencies in microsurgery, through the verification of the average score achieved in the OSATS, by the urology residents, during the training course. In this image, a progressive and significant increase can be seen, from a statistical point of view, in the score throughout the weekly training sessions, reaching a plateau in S4, and showing a slight drop in performance in S5 (p < 0.0001).

Table 1 expresses the SSSCL score, which was applied to participants at the end of the course. Its analysis confirms the residents’ satisfaction with the learning obtained during this training period, with general approval rate of 96.9%, with a Cronbach’s alpha coefficient of 83%.

*Analysis of variance: p = 0.0001. Source: Elaborated by the authors.

**Table 1:** Scale of Student Satisfaction and Self Confidence in Learning by urology residents after the Training Course in Urological Microsurgery.

Evaluation domains	Evaluation		Cronbach’s index
	Score	% Approval	
Satisfaction with currenting learning	197	98,5	0,856
Self-confidence in learning	307	95,9	0,807
Overall rating	504	96,9	0,832

Source: Elaborated by the authors.

## Discussion

According to the competency matrix of medical residency programs in urology in Brazil, prepared in 2018 by the Ministry of Education, in partnership with the Brazilian Society of Urology, residents in this specialty, at the end of their training, must be able to carry out a series of medium- and high-complexity procedures, including understanding the pathophysiology, diagnosis and treatment of infertility and male hypogonadism, as well as being able to perform surgical techniques that require cutting-edge technology, such as vasovasostomy, vasoepididymal anastomosis and microsurgical correction of varicocele^1^.

This study was conducted by the UEPA, which is responsible for maintaining the two urology residency programs in the region, annually training three new professionals to meet the demands of the Amazonian population. Unfortunately, like most health education institutions in Brazil, we have evidenced some gaps in the training of these professionals, especially in the sub-areas of urology that are more dependent on high-cost equipment, such as microsurgery^4^^,^ 5 ^,^ 8. This lack of training for residents, as well as an increasing demand from patients seeking to treat or recover their fertility, was the main motivator for the creation of a line of research at the university involving microsurgery and urology.

Although there is no universal standardization, most studies involving training in microsurgery choose to conduct training courses, containing five practical sessions^17^, which was followed in this work. We believe that sessions with weekly breaks are more productive compared to intensive courses daily. The classic study by Moulton et al. suggests that, during training intervals, different regions of the brain become activated, each considered necessary for permanent retention of surgical skills and competencies. The fact of having to search the memory for key aspects of the skill that is being learned helps to solidify this skill more deeply in the memory^18^^,^ 19.

The literature has demonstrated several ways of assessing performance during training sessions, which involve the use of checklists, scales, and performance measurement through motion sensors, among others^18^^,^ 20. We believe that the measurement of suture-making time should not be used in isolation as a performance verification tool, given that making quick knots does not mean the same thing as well-made knots. Thus, this assessment tool must be accompanied by another form of performance quantification. In this study, we opted for the OSATS, which is the global assessment scale, most used for this purpose, and which was recently validated for the Portuguese language^14^.

For Ghanem et al., the use of the global rating scales in these assessments has the main objective of increasing the objectivity and quantification of performance, reducing the subjective effect of this assessment. In addition, another aspect of the immense value of this tool is that it allows the residents who participated in the study to monitor their evolution, facilitating during the debriefing the identification of the points that needed to be improved for the next training sessions^21^.

The implementation of the SSSCL aimed to seek feedback from urology residents on their perception, as well as the degree of satisfaction, concerning the learning obtained at the end of the training course in microsurgery. This tool was validated for Portuguese in 2015 and we used it because we believe that the resident’s well-being and the self-confidence gained through learning are important constructs in the work environment, and knowing how to measure them can provide us with valuable information for structuring teaching plans. In the present study, the results found were quite encouraging, with a general satisfaction rate of 96.9%, which has been encouraging us to idealize other simulators, to collaborate in the teaching-learning of other areas of urology, in which our residents also have some gaps identified in their training, such as, for example, in the endoscopic management of urolithiasis^3^.

The scientific literature has shown that the use of low-fidelity artificial simulators has been used in an increasingly common way, as an auxiliary tool in the teaching-learning process in surgical medical residencies, with descriptions of training models that vary from synthetic models, vegetables, fruits, parts of animals, among others^22^^,^ 23. According to Evgeniou et al., the use of such experimental models has the following main positive points: they are very accessible, have low cost, allow repeated training several times, without the need to sacrifice experimental animals, in addition to facilitating the training of residents in existing gaps between his busy schedule of outpatient care, visits to the ward and procedures in the operating room^24^.

The results of this research demonstrated that training using a low-fidelity simulator was able to promote the gain of skills and competencies in microsurgery by urology residents. This was identified in practical sessions by the elaboration of faster microsurgical sutures, in pre- and post-training, as well as by the global improvement in the creation of microsurgical anastomosis, verified by the progressive increase in the values of the OSATS, with both findings being relevant from a statistical point of view, with p < 0.05. Another important aspect to be emphasized is that this was the first study on the subject that sought to verify the long-term retention of practiced knowledge, using low-fidelity simulators. Our results demonstrated that, despite the drop in performance on the part of the residents, in the last training session, the time for making the sutures, as well as the OSATS values, remained within a plateau, suggesting a persistent stability in the acquired skills and competences, persisting even after a three-month interval between S4 and S5.

For Crouch et al. the objectives intended with the simulation are divided into four non-exclusive domains^25^, which are:

- Use of specific equipment;
- Performance of certain manual movements;
- Recognition and familiarity with anatomical locations;
- Replication of a surgical procedure in its entirety.

This author identified in his study that most low-fidelity simulators described in the literature cover at least two of these domains, ensuring the gain of expertise in microsurgery at basic and intermediate levels^25^. In our understanding, we argue that this expertise in microsurgery was achieved by the study participants. However, for the acquisition of more advanced skills, it would be necessary to use high-fidelity models.

Most publications still advocate the use of experimental animals, most rats, as the gold standard for training in microsurgery^26^. According to Gasteratos et al.^26^, the use of live animals in microsurgery training is far superior to other training modalities, not only because of the manipulation of organic and physiological structures more similar to that of humans, but also because it is possible to acquire skills and extra, non-technical skills, such as the ability to make quick decisions; learn to deal with surgical stress when operating on a living organism; handling potential intraoperative complications that may arise unexpectedly; among others.

This is the main limitation of this work. As the training sessions took place only with a synthetic simulator, the structures elaborated in 3D printing do not have the same consistency as the real vas deferens, not allowing, for example, the performance of more elaborate anastomoses, in multiple planes, as well as the perfect coaptation of their stumps. However, the results of this study make it noticeably clear that artificial simulators can guarantee the gain of basic and even intermediate microsurgical skills and competencies; and that, by itself, is already a facilitator for the future acquisition of advanced expertise. This is corroborated by the findings by Lahiri et al., which demonstrated that it is possible to maintain high-quality microsurgical training, similar to those involving exclusively laboratory animals, considerably reducing the number of rats, especially in the initial stages of training, replacing them with synthetic models^27^.

Our institution has also been following the policy of the three Rs, involving animal experimentation (reduction in the number of animals used; replacing with other experimental models, and refinement of artificial models)^26^^,^ 27. An alternative that we have found for this resides in the use of parts of slaughtered animals, little used in gastronomy, and which are normally discarded, such as pig kidneys and testicles. These organs closely resemble those of humans, ensuring high-fidelity microsurgical training. In addition, their jobs eliminate the need for the study to be released by the Ethics Committee on Animal Use. The microsurgery group at the UEPA has been using this alternative, with very encouraging initial results^28^^,^ 29.

This study was a continuation of the work originated in 2019, aiming at ways to qualify urologists trained in the Amazon region. At that time, the publication generated a long editorial comment, praising the potential of the idea, but also making some criticisms and suggestions, which basically involved the method of quantifying performance (through a checklist without prior validation) and, mainly, asking questions regarding the ability of low-fidelity simulation to guarantee lasting retention of skills and competencies in microsurgery.

The manuscript in question differs from the 2019 article, which focused much more on the quality of the training offered to our residents, rather than on the simulator itself, and in that we sought to answer the main questions raised in that editorial comment. The main points highlighted are: the use of almost double the number of participants (nine in total), covering the two medical residency services in urology in the state of Pará; the quantification of residents’ performance with the OSATS, a tool validated for Portuguese only in 2020, considered much more robust than a checklist and specifically focused on the nuances of microsurgery (delicacy in handling the structures, degree of force used, appropriate use of instruments, among others); the feedback and degree of satisfaction of urology residents with the training offered (through the SSSCL) and, mainly, the analysis of the retention of skills and competencies in microsurgery, months after the implementation of the low-fidelity simulation.

Finally, we suggest for further publications articles involving the manufacture of more artificial simulators, as well as evaluation tools, specifically aimed at urological microsurgery, such as, for example, in qualifying for the management of varicocele, vasectomy reversal, penile reimplantation, etc. The literature is still lacking in this type of information, making it necessary to use data from studies in the field of plastic surgery, orthopedics, and neurosurgery.

## Conclusion

The low-fidelity simulation was able to guarantee urology residents a solid gain in skills and competencies in microsurgery.

## Data Availability

All data sets were generated or analyzed in the current study;
